# Protective effect of dexmedetomidine against delayed bone healing caused by morphine via PI3K/Akt mediated Nrf2 antioxidant defense system

**DOI:** 10.3389/fphar.2024.1396713

**Published:** 2024-05-28

**Authors:** Yani Lou, Linfang Zou, Zhenyu Shen, Jianwei Zheng, Yuanqu Lin, Zhe Zhang, XuanKuai Chen, Jun Pan, Xutong Zhang

**Affiliations:** ^1^ Department of Anesthesiology and Perioperative Medicine, The Second Affiliated Hospital and Yuying Children’s Hospital of Wenzhou Medical University, Wenzhou, China; ^2^ Key Laboratory of Anesthesiology of Zhejiang Province, Wenzhou Medical University, Wenzhou, China; ^3^ Department of Orthopaedics, The Second Affiliated Hospital and Yuying Children’s Hospital of Wenzhou Medical University, Wenzhou, China

**Keywords:** morphine, oxidative stress, dexmedetomidine, calvarial defect, PI3K/Akt/Nrf2 pathway

## Abstract

**Background:**

As a class of analgesics, opioids are frequently used to treat both acute and chronic moderate to severe pain. Patients frequently receive opioid painkillers after orthopedic accidents or surgeries. Evidence suggests that opioid drug users have a 55.1% higher risk of fracture and poor bone repair than non-users of opioid drugs. The key pathogenic alterations in the incidence and progression of poor bone repair are over apoptosis and aging of osteoblasts due to the stress caused by oxidation. Dexmedetomidine (Dex) has been proven to protect against a variety of degenerative illnesses by reducing oxidative stress. However, nothing is known about how it affects bone repair.

**Methods:**

PI3K/Akt/Nrf2 pathway was detected by immunofluorescence and Western blot. SOD, CAT, JC-1, dihydroethidium and mitosox were used in the Oxidative Stress. Micro-CT, H&E and Masson’s staining, immunohistochemically were performed to evaluate the therapeutic effects of DEX on calvarial defects in the morphine-induced rat model.

**Results:**

We found that morphine-induced an imbalance in the metabolism and catabolism of primary rat Osteoblasts. However, these conditions could be inhibited by DEX treatment. In the meantime, DEX induced the expression of Nrf2-regulated antioxidant enzymes such as NQO1, HO-1, GCLm, GCLc, and TrxR1. DEX-mediated Nrf2 activation is linked to the PI3K/Akt signaling system. Furthermore, it has been established that intravenous DEX enhanced the growth of bone healing in a model of a surgically produced rat cranial lesion.

**Conclusion:**

This is the first description of the unique DEX mechanism acting as a Nrf2 activator against morphine-mediated oxidative harm, raising the possibility that the substance may be used to prevent bone defects.

## 1 Introduction

One of the best opioid analgesics for treating severe acute and chronic pain is morphine (Sverrisdóttir et al., 2015). The analgesic withdrawal symptoms of morphine, which are primarily mediated by μ-opioid receptors, are mediated by numerous types of signaling mechanisms (Zhuang et al., 2022). However, a number of unwanted side effects, including headache, gastrointestinal issues, cough suppression, or respiratory depression, come along with these beneficial antinociceptive benefits (Tiseo et al., 1995). Evidence suggests that compared to non-users of opioids, opioid users had a 55.1% higher risk of fracture and poor bone repair (Saunders et al., 2010; Coluzzi et al., 2020). Numerous studies imply that oxidative stress may contribute to the onset of these unfavorable occurrences (Cai et al., 2016). Reactive oxygen species (ROS), which are created when oxygen is partially reduced, are produced and degraded in an unbalanced manner during oxidative stress events (Filomeni et al., 2015). According to several studies, morphine increases the process of creation of ROS and decreases the activity of several antioxidant-producing enzymes (Reymond et al., 2022; Taghavi et al., 2023).

The cellular antioxidant defense system is heavily dependent on the Nuclear factor (erythroid-derived 2)-like 2 (Nrf2), an omnipresent regulator of the antioxidant consequence (Kobayashi et al., 2016). Numerous research have been carried on to uncover Nrf2-downstream target genes, which involve antioxidant phase II detoxifying enzymes, despite the Nrf2-dependent antioxidant reaction being an intricate in addition well-structured cellular mechanism (Zhang et al., 2013). Numerous signaling mechanisms, including nuclear localization and nuclear rejection indications, control the nuclear accumulation of Nrf2 (Wang et al., 2022). The phosphatidylinositol 3-kinase (PI3K)/Akt pathway has been identified as a key upstream controller of Nrf2 nuclear localization and controls a broad range of processes inside cells, which include proliferation, advancement, differentiation, and movement (Yu and Xiao, 2021). Phosphoinositide-dependent protein kinase-1 phosphorylates and triggers Akt once PI3K has been triggered by a variety of stimuli, which then causes the stimulation of Nrf2 and the induction of Nrf2-mediated production of antioxidant/phase II detoxification enzymes (Su et al., 2012).

DEX is a highly selective α_2_-adrenoceptors agonist (AR), it has sedative, analgesic,opioid-sparing, sympatholytic effects (Keating, 2015). DEX also has anti-apoptotic effects and inhibits the production of inflammatory mediators in patients with craniocerebral injury. These actions effectively increase vascular stability, lessen cerebral edema brought on by craniocerebral injury, and improve perioperative brain function in ischemic attack (IA) patients (Benggon et al., 2012; Burlacu et al., 2022). Numerous studies have documented how DEX reduces oxidative stress to protect against a variety of degenerative illnesses (Wang et al., 2020; Shi et al., 2021). The therapeutic effects of DEX on bone abnormalities as a condition brought on by oxidative stress, however, have not been shown. The main objective of this research investigation was to figure out how DEX influences the bone-healing process.

## 2 Materials and methods

### 2.1 Antibodies and reagents

Abcam (Cambridge, MA, United States) provided the COL1A1, RUNX2, OCN, PI3K, p-PI3K, and GAPDH; and ProteinTech (Wuhan, China) provided the primary antibodies against Nrf2, NQO1, GCLc, and Gclm. The antibodies were purchased from Cell Signaling Technologies (Danvers, MA, United States) and were directed against P-Akt, Akt, TrxR1, Keap1, and HO-1. Fetal bovine serum (FBS), Dulbecco’s Modified Eagle Medium (DMEM), and penicillin/streptomycin were purchased from Gibco BRL (Thermo Fisher Scientific, Waltham, MA, United States). The criteria for tissue and cell culture were followed by all other substances, which were all of analytical quality.

### 2.2 Isolation and primary culture of osteoblasts

One-day-old Sprague Dawley rats’ calvarial bones were used to separate primary osteoblasts, which were then grown in full DMEM complemented with 10% (v/v) FBS and 1% (v/v) penicillin-streptomycin under 5% CO2 at 37°C. (Jonason and O'Keefe, 2014). The cells were transmitted when they amounted to 80%–90% intersection, and the medium was replaced every other day. Cells underwent the following therapy, in brief: 1) Morphine group: cells were cultured in full DMEM media for 2 h after being exposed to 100 μM morphine for 48 h 2) Morphine + DEX group: cells were exposed to 100 μM DEX for 48 h, then co-cultured with 0.1 and 1 μM DEX for 2 hours. 3) Control group: Osteoblasts that had not been given any treatment were cultivated for a comparable period.

### 2.3 Animal model

At the Shanghai Animal Center of the Chinese Academy of Sciences, 46 male Sprague-Dawley rats were acquired. They were given unlimited access to food and drink while being kept in an SPF environment with a 12-h light/12-h dark cycle. The Wenzhou Medical University Animal Research Committee approved all tests using rats, and the surgical procedures followed the guidelines established by the Ethics Committee for Animal Research (Animal Ethics Number:WYDW 2020-0564). After acclimation of the rats for 1 week, they were divided into three groups.: control, morphine, and DEX + morphine. The rats were subsequently rendered unconscious by an intraperitoneal dose of 50 mg/kg pentobarbital sodium. The dissection was carried down to the calvarium after a 1.5 cm incision was performed in the scalp. A 5-mm-diameter trephine was used to make a critical-size calvarial defect after the periosteum was removed, with saline water being given for cooling. For 12 weeks following craniotomy, rats in the DEX + morphine group received DEX injections at 7 mg/kg using a 5 L microinjector (flat tip diameter, 0.3 mm; needle length, 2.5 cm). A single dosage of 100 mg/kg of morphine hydrochloride was slowly injected into the rats in the morphine group.

### 2.4 Western blot assay

With the use of the RIPA lysis solution containing 1 mM PMSF (phenylmethanesulfonyl fluoride), the total protein in the osteoblasts was extracted. The BCA protein assay kit (Beyotime) was used to calculate the protein concentration. On sodium dodecyl sulfate-polyacrylamide gel electrophoresis (SDS-PAGE) gels, the protein (40 ng) was separated, and then it was transferred to a polyvinylidene difluoride (PVDF) membrane. Following a 2-h blocking step in which the membrane was incubated with 5% non-fat milk, the membrane was then incubated overnight at 4°C with the primary antibodies COL1A1, NRF2, RUNX2, OCN, PI3K, p-PI3K, GAPDH, NQO1, GCLc, Gclm, HO-1, P-Akt, Akt, TrxR1, and Keap1. The bands were then identified using the matching secondary antibodies and an electrochemiluminescence reagent (Invitrogen) for 2 hours at room temperature. This was done after three TBST washes. The intensity of blots was then calculated using Image Lab 3.0 software (Bio-Rad).

### 2.5 Immunofluorescence

Six-well plates with glass coverslips were used to seed osteoblasts, which were then washed with PBS, fixed in 4% paraformaldehyde, and permeated with 0.1% Triton X-100 for 15 min. The osteoblasts were treated overnight at 4°C with the primary antibody against Nrf2 (1:200) following blocking with 5% bovine serum albumin for 30 min. The cells were washed the next day and labeled for 1 h at room temperature using Alexa Fluor 488 or Alexa Fluor 594. The slides were examined with a confocal scanning microscope (Nikon, Japan), and ImageJ software 2.1 (Bethesda, MD, United States) was used to evaluate the fluorescence intensity.

### 2.6 Osteogenic differentiation of Primary Calvarial Osteoblasts

Primary Calvarial Osteoblasts’ Osteogenic Differentiation. A 24-well plate containing the cells was planted with 5 104 cells per well. Osteoblasts were grown in DMEM with 20 mM ascorbic acid and 10 mM -glycerophosphate after receiving the recommended therapy. Every other day, the media was switched out. After 7 days of differentiation, the alkaline phosphatase (ALP) activity was assessed using the ALP Staining Kit from the Beyotime Institute of Biotechnology in Jiangsu, China. The cells were grown in osteogenic conditions for 21 days to induce mineralization and the development of bone nodules. They were then fixed and stained with Alizarin Red S (ARS) by Solarbio Science and Technology, Beijing, China.

### 2.7 Quantification of SOD and CAT activities

The appropriately treated cells were lysed on ice for 30 min after being washed twice with PBS. According to the manufacturer’s instructions, commercial assay kits (Jiancheng Biotechnology, Nanjing, China) were used to measure the activity of SOD and CAT in the lysates.

### 2.8 Intracellular ROS assay and mitochondrial function assays

Reactive oxygen species within cells were measured using a dihydroethidium (DHE) probe (Yeasen, Shanghai, China). According to the manufacturer’s instructions (Beyotime, Shanghai, China), the amounts of superoxide ions and mitochondrial membrane potential (MMP) in the properly treated osteoblasts were measured by, respectively, staining with JC-1 and MitoSox. A fluorescent microscope (Olympus Life Science; Tokyo, Japan) and confocal scanning microscopy (Nikon; Japan) were used to examine stained cells.

### 2.9 Micro-CT analysis

Utilizing a cabinet conebeam micro-CT system and related software (CT 50, Scanco Medical; Brüttisellen, Switzerland), a microstructural investigation of the calvarial defect was carried out. The photos were taken at a voltage of 70 kV, an amperage of 200 A, and a spatial resolution of 14.8 mm all around. The trabecular compartment 2 mm below the greatest point of the growth plate to the distal 100 CT slices was included in the volume of interest (VOI), which was used to create three-dimensional reconstructed images. The ratio of bone volume to tissue volume (BV/TV), the mean trabecular number (Tb. N, 1/mm), the mean trabecular thickness (Tb. Th, mm), the mean connective density (Conn.D, 1/mm3) and the mean trabecular separation (Tb. Sp, mm) were among the quantitative bone characteristics evaluated inside the VOI.

### 2.10 Histopathologic analysis

After removing the dependent tissues, the calvarial defect tissues were collected and preserved with 4% formaldehyde for 48 h. The samples were then decalcified for a month at 4 °C using a 10% ethylenediamine-tetraacetic acid (EDTA) solution. 70%–100% ethanol gradient dehydration, xylene clearing, and paraffin embedding. According to the manufacturer’s directions, longitudinal 4 m-thick serial slices were stained with hematoxylin-eosin and Mason trichrome. For IHC, 6-m-thick sections were treated with the anti-VEGF, CD31 primary antibody before being subjected to the manufacturer’s recommended horseradish peroxidase detection procedure (Vector Laboratories; Burlingame, CA, United States).

### 2.11 Statistical analysis

The findings have been laid out as mean ± SD. The statistical application SPSS 18.0 was used to examine the data. ANOVA or t-tests were used to evaluate whether there were differences between the groups. At P 0.05, statistical differences within and/or between groups were taken into consideration. Three separate runs of each experiment showed that they could all be reliably repeated.

## 3 Results

### 3.1 Effects of morphine and/or DEX treatment on rats’ osteoblasts viability

**FIGURE 1:**
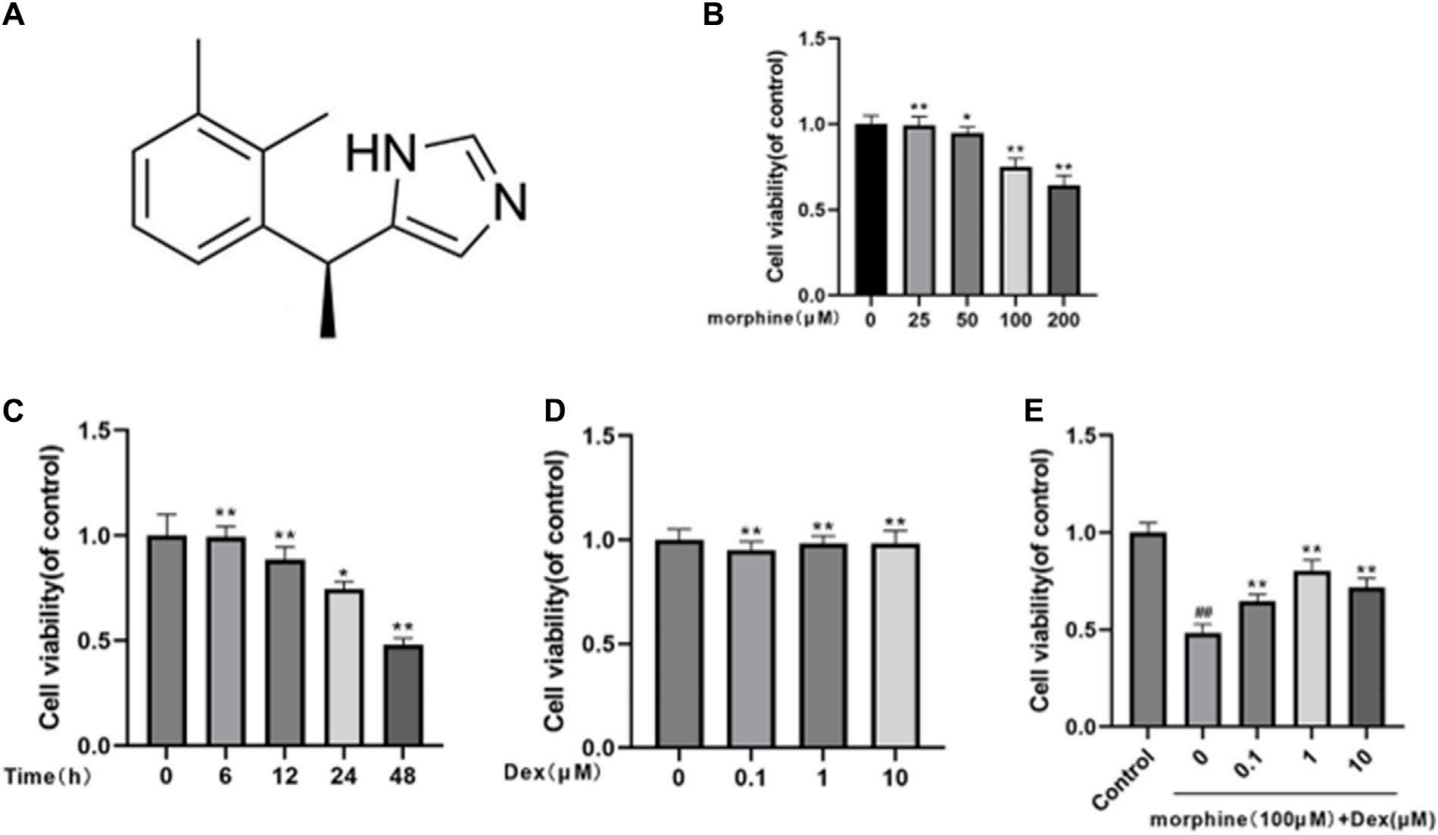
Effects of morphine and/or DEX administration on the viability of rat osteoblastic cells. **(A)** The dexmedetomidine chemical structure. **(B)** The percentage of viable cells treated for 24 h with various dosages of morphine. **(C)** Morphine treatment percentage is given to live cells throughout a time course of 0, 6, 12, 24, and 48 h at a 100 µM concentration. **(D)** How various DEX dosages affect cell viability. **(E)** The impact of morphine on cell survival when combined with various DEX concentrations. Data were evaluated as mean + SD. *p < 0.05, **p < 0.01 compared to the control group; #*p* < 0.05, ##*p* < 0.01, compared to the morphine stimulation group, *n* = 3.

Figure 1A depicts DEX’s chemical make-up. The CCK8 test was used to investigate the effectiveness of morphine and/or DEX in preserving osteoblast viability. To determine the proper dose and duration of the stimulus, osteoblasts were first given morphine in a dose-dependent manner (0, 25, 50, 100, 200 μM) for 24 h and in a time-dependent manner (0, 6, 12, 24, 48 h) at 100 μM (Figures 1B, C). At a dosage of 100 μM morphine for 48 h, it was found that cell viability was significantly decreased (survival rate <50%). For the following investigations, 100 μM morphine with a 48 h treatment duration was used. The effects of DEX on the cell, whether or not it was combined with morphine, were then also investigated. CCK-8 tests were used to determine how DEX affected cell viability. The findings showed that, in comparison to exposure to 0 μM DEX, there was no significant difference in cell viability when Osteoblasts were subjected to 0.1–10 μM DEX (Figure 1D). The CCK-8 results indicated that the safeguarding effect of DEX towards morphine-induced cytotoxicity was best at the concentration of 1 μM after the cells had been treated with 100 μM morphine and various doses of DEX (Figure 1E). The results mentioned above imply that DEX corrected the reduction in cell viability caused by morphine.

### 3.2 DEX reestablished the mineralization and differentiation of morphine-treated osteoblasts

**FIGURE 2:**
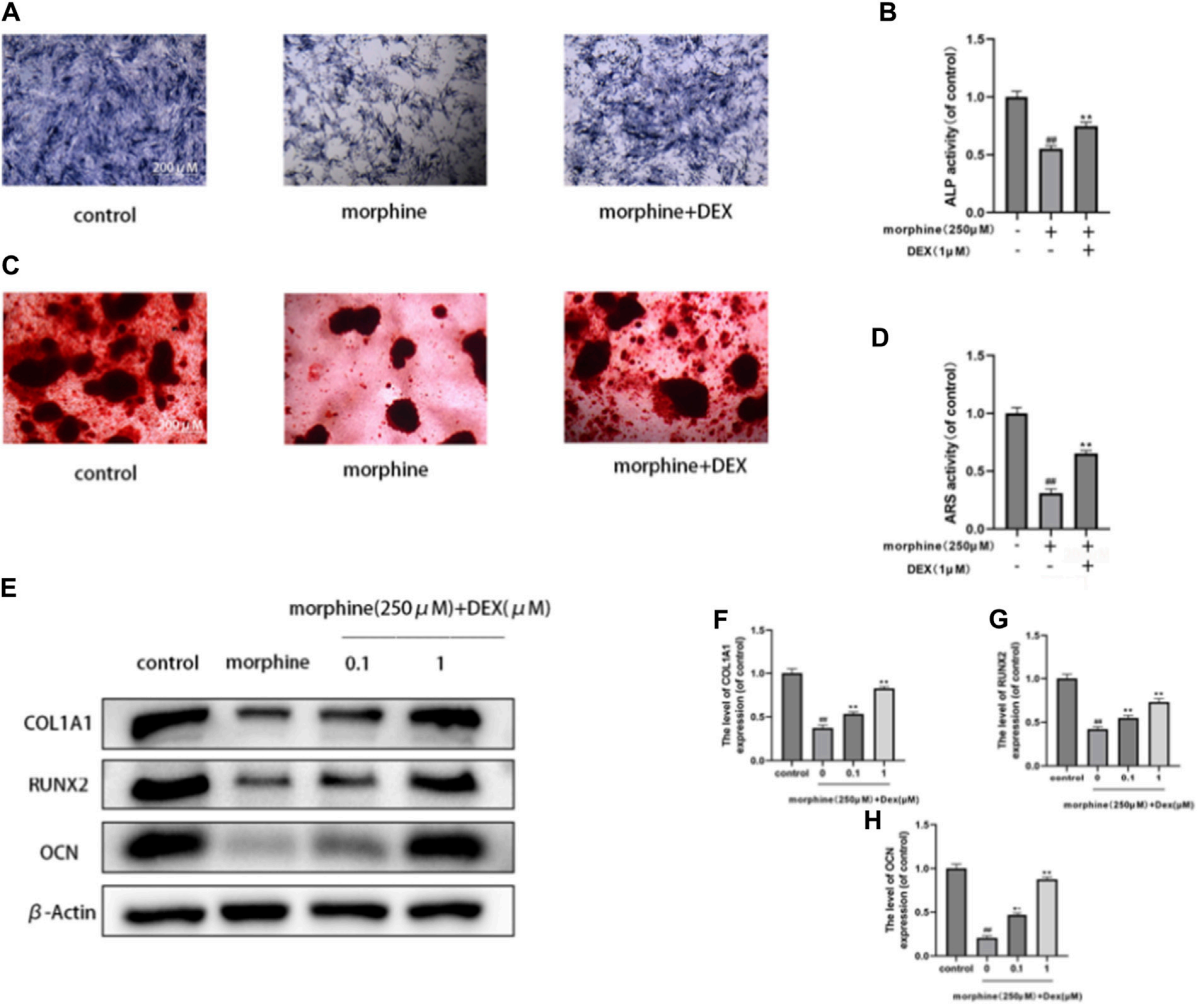
DEX reestablished the mineralization and differentiation of morphine-treated osteobiasts. **(A–D)** Differentially treated osteoblasts ALP stained on the 7th day and ARS stained on the 21st day after osteogenic induction. DEX restored differentiation and mineralization. **(E–H)** OCN, RUNX2, and COL1A1 expression levels in the variously treated osteoblasts. Data were evaluated as mean +SD. *p < 0.05, **p < 0.01 compared to the control group; #*p* < 0.05, ##*p* < 0.01, compared to the morphine stimulation group, *n* = 3.

Osteoblasts’ capacity for bone formation is compromised by oxidative stress and mitochondrial malfunction. Next, we evaluated how DEX affected the morphine-treated cells’ early differentiation and mineralization. Morphine significantly lowered calcium nodule development by the 21st day (Figures 2A–D) and significantly inhibited osteogenic differentiation, as seen by the ALP activity following a 7-day culture. After 7 days of osteogenic induction, cotreatment with DEX increased the levels of the osteogenic transcription factors OCN, COL1A1, and RUNX2, which also restored ALP activity and the degree of mineralization in the morphine-treated osteoblasts (Figures 2E–H). When combined, DEX shields osteoblasts from morphine’s inhibitory effects.

### 3.3 DEX neutralized morphine-induced oxidative stress in osteoblasts

**FIGURE 3:**
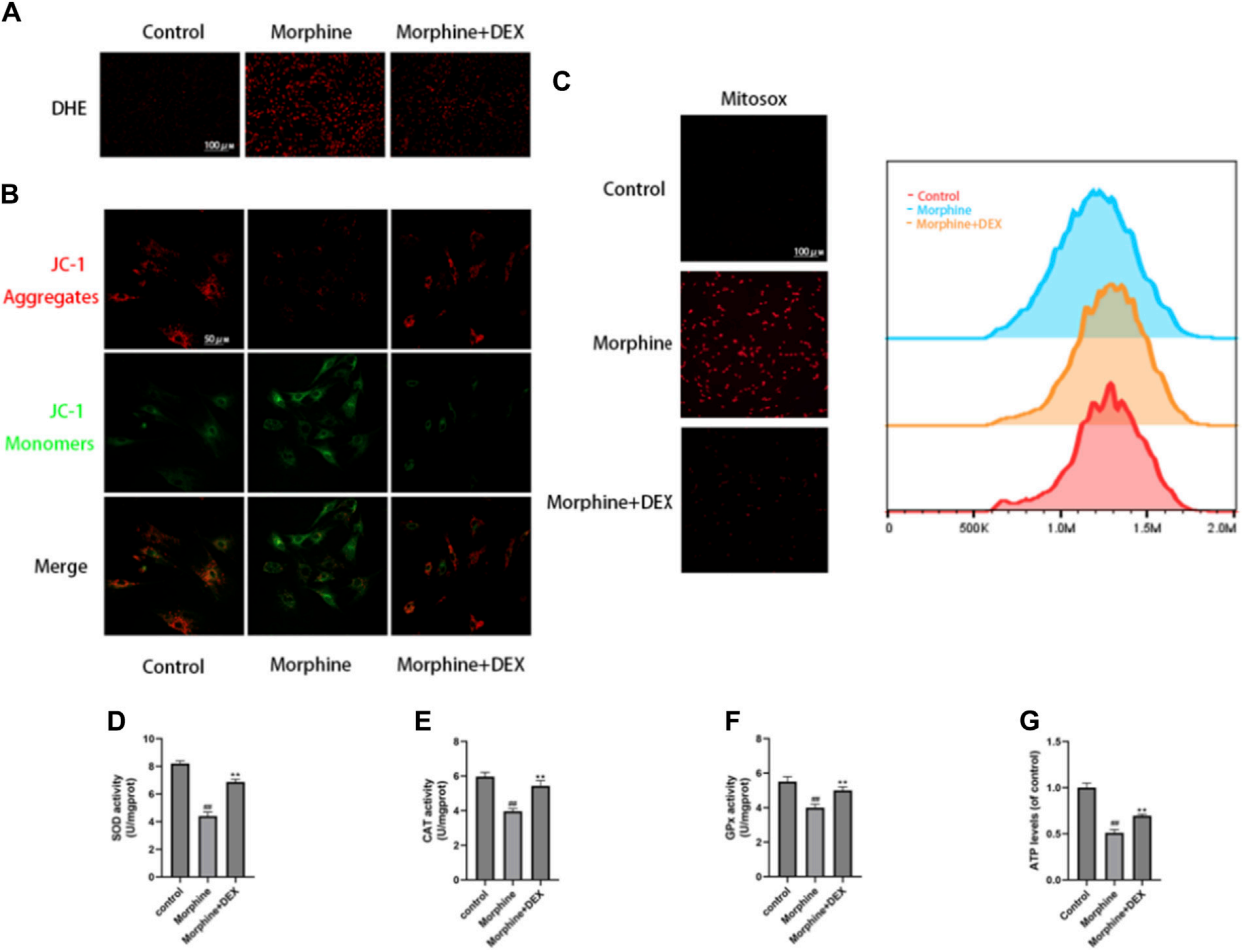
DEX Reduced Oxidative Stress in Osteoblasts Caused by Morphine. **(A)** Images of the DHE stains tnat are representative. **(B)** Confocal fluorescence microscopy was used to identify JC-1 aggregates (red) and monomers (green) in osteoblasts. **(C)** MitoSox (red) was used for mtROS staining. MitoSox (red) staining representative photos are displayed as the relative mean fluorescence intensity as determined by flow cytometry. **(D–F)** The activities of GPx, CAT, and SOD in cells treated with morphine with or without DEX. **(G)** The osteoblasts received various treatments and their ATP levels. Data were evaluated as mean +SD. *p < 0.05, **p < 0.01 compared to the control group; #*p* < 0.05, ##*p* < 0.01, compared to the morphine stimulation group, *n* = 3.

The primary pathogenic changes in delayed bone repair are increased apoptosis and senescence of osteoblasts caused by oxidative stress. We thus assessed the effects of DEX on the generation of ROS, dysfunction of the mitochondria, and the functioning of antioxidant enzymes in the morphine-treated osteoblasts. Using the DHE fluorescence to analyze intracellular ROS, it was shown that morphine administration enhanced ROS buildup, which was countered by DEX pretreatment (Figure 3A). JC-1 and MitoSox probes were used, respectively, to identify the MMP and mitochondrial ROS. In comparison to the control cells, the morphine-treated cells exhibited considerably decreased MMP and higher amounts of superoxide anion, both of which were nearly restored to normal levels by DEX administration (Figures 3B, C). Along with restoring ATP levels, morphine-treated osteoblasts cocultured with DEX also restored the activities of CAT, GPx, and SOD (Figures 3D–G).

### 3.4 Effects of DEX on Nrf2 nuclear localization

**FIGURE 4:**
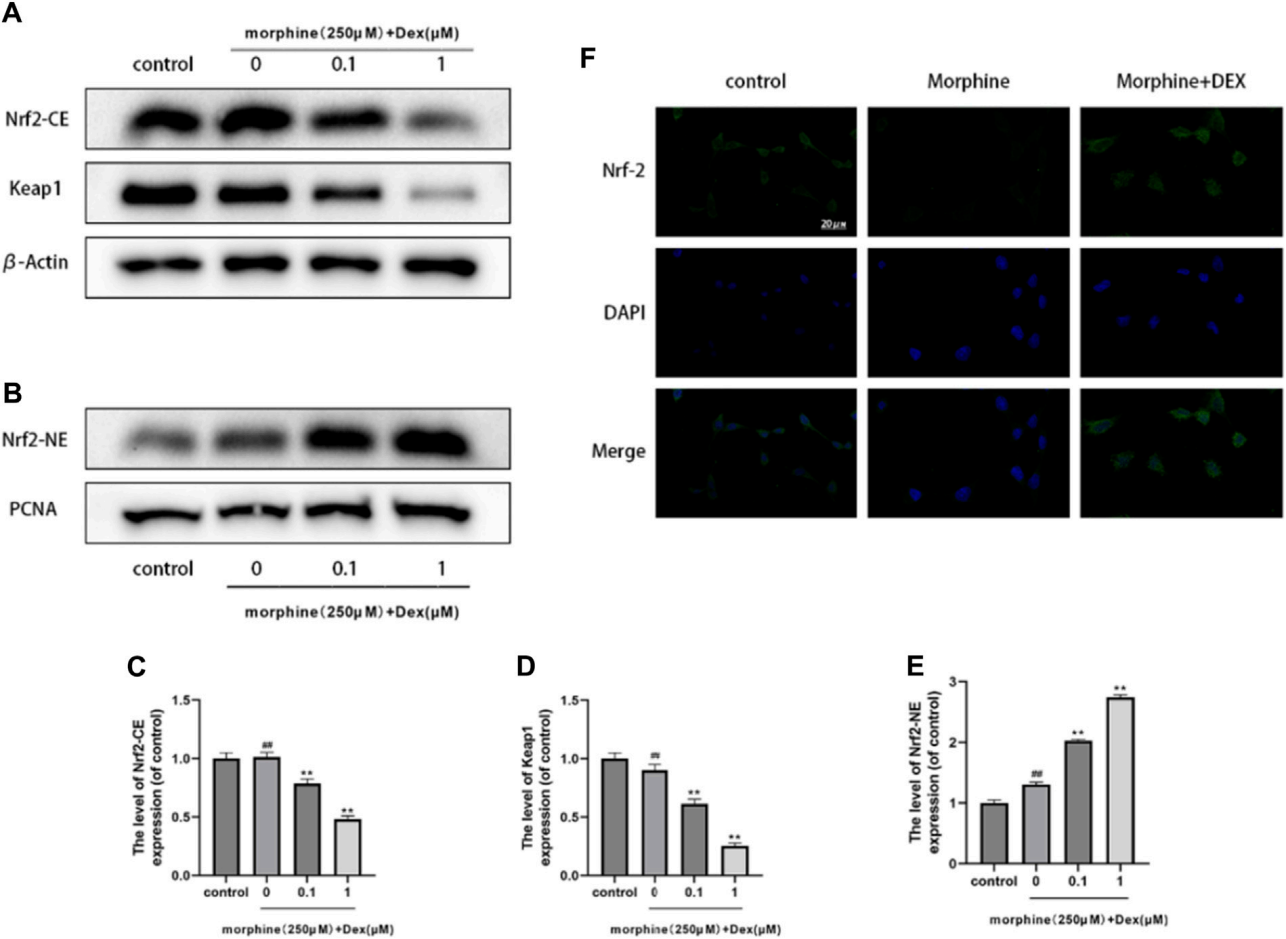
Effects of DEX on Nrf2 nuclear localization **(A–E)**. Immunoblot demonstrating Nrf2-CE, Nrf2-NE, and Keap1 levels in the variously treated osteoblasts. **(F)** Typical fluorescence pictures displaying Nrf2 localization in the cells that underwent different treatments. Data were evaluated as mean +SD. *p < 0.05, **p < 0.01 compared to the control group; #*p* < 0.05, ##*p* < 0.01, compared to the morphine stimulation group, *n* = 3.

The expression of Intracellular Nrf2 and Keapl was examined to establish DEX as the powerful Nrf2 activator and to demonstrate Nrf2 nuclear accumulation by DEX. Keapl typically locks Nrf2 away in the cytoplasm, however when Nrf2 is let out into the nucleus, antioxidant/phase II detoxification enzymes can be activated. The level of intracellular Nrf2 expression did not change after morphine treatment, as shown in Figure 4A (*p* 0.05). However, DEX significantly decreased cytoplasmic Keapl expression at all doses (Figure 4A). The nuclear translocation of Nrf2 was examined to identify the strong Nrf2 promoter as a need for stimulation of the endogenous protective antioxidant system. As seen in Figures 4B–E, DEX caused a more than threefold increase in Nrf2 nuclear accumulation whereas morphine had no impact on Nrf2 translocation. The findings of the immunofluorescence labeling showed that DEX co-administration considerably increased the expression of Nrf2 in comparison to the morphine-treated cells (Figure 4F). These findings indicate that Nrf2 activation is linked to DEX’s protective effect against morphine-induced oxidative damage.

### 3.5 DEX’s effects on phase-II detoxification enzymes and antioxidants

**FIGURE 5:**
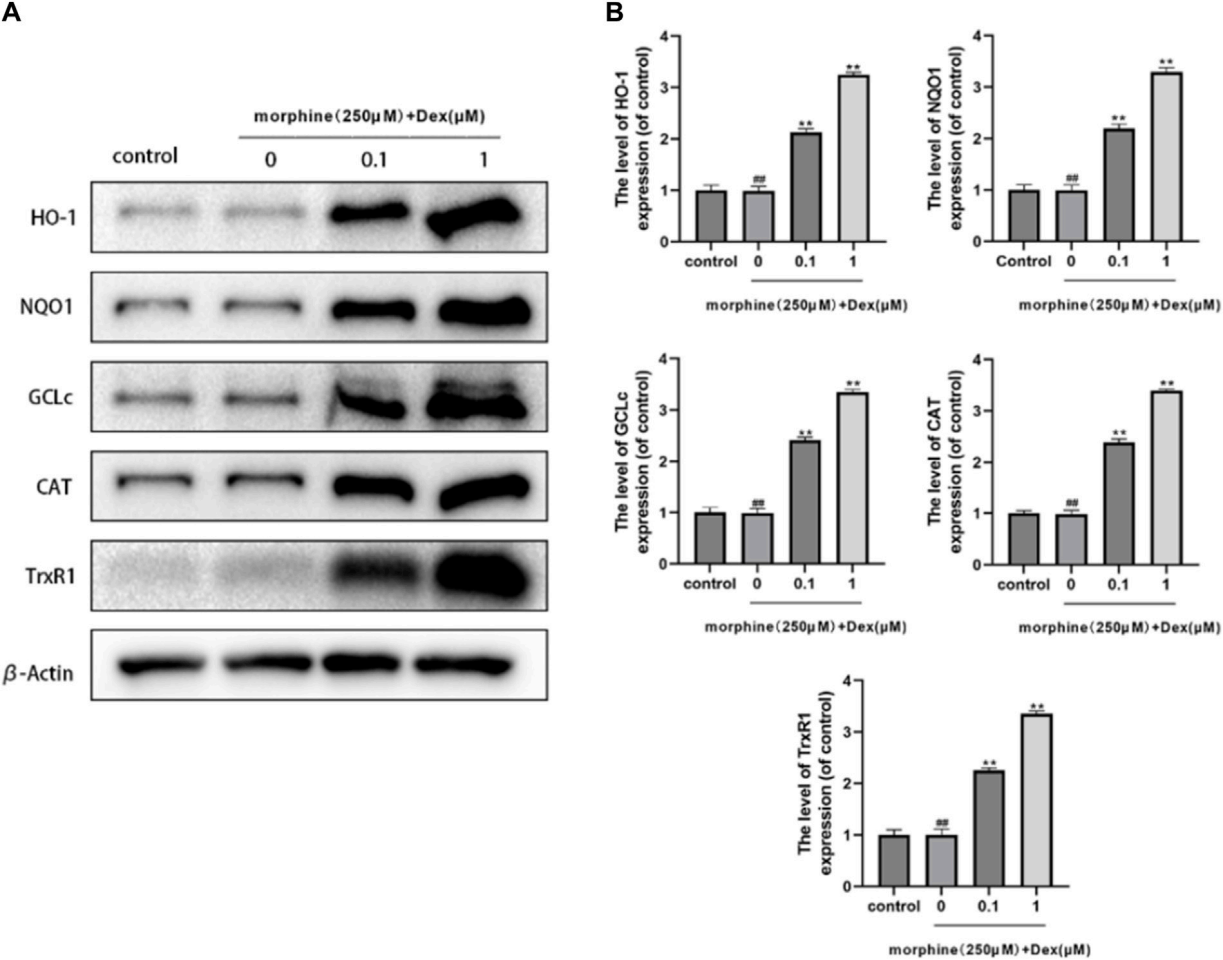
Effects of DEX on antioxidant/phase-I| detoxification enzymes. **(A,B)** The protein expression of HO-1, NQO1, GCLc, CAT, and TrxR1 is shown in the osteoblasts treated as described above. Data were evaluated as mean +SD. *p < 0.05, **p < 0.01 compared to the control group; #*p* < 0.05, ##*p* < 0.01, compared to the morphine stimulation group, *n* = 3.

The production of antioxidant/phase II detoxifying enzymes, Nrf2’s downstream target, provided evidence of the transcriptional action of Nrf2. DEX had an impact on the expression of Nrf2-driven antioxidant/phase II detoxification enzymes such as GCLc, CAT, TrxR1, HO-1, and NQO1. When compared to the morphine-treated group, the expression of HO-1, NQO1, GCLc, CAT, and TrxR1 was dramatically increased more than two times, demonstrating that DEX is a powerful trigger of Nrf2-driven antioxidant responses (Figure 5).

### 3.6 Impacts of DEX on the downstream antioxidant enzymes and the PI3K/akt/Nrf2 signaling pathway

**FIGURE 6:**
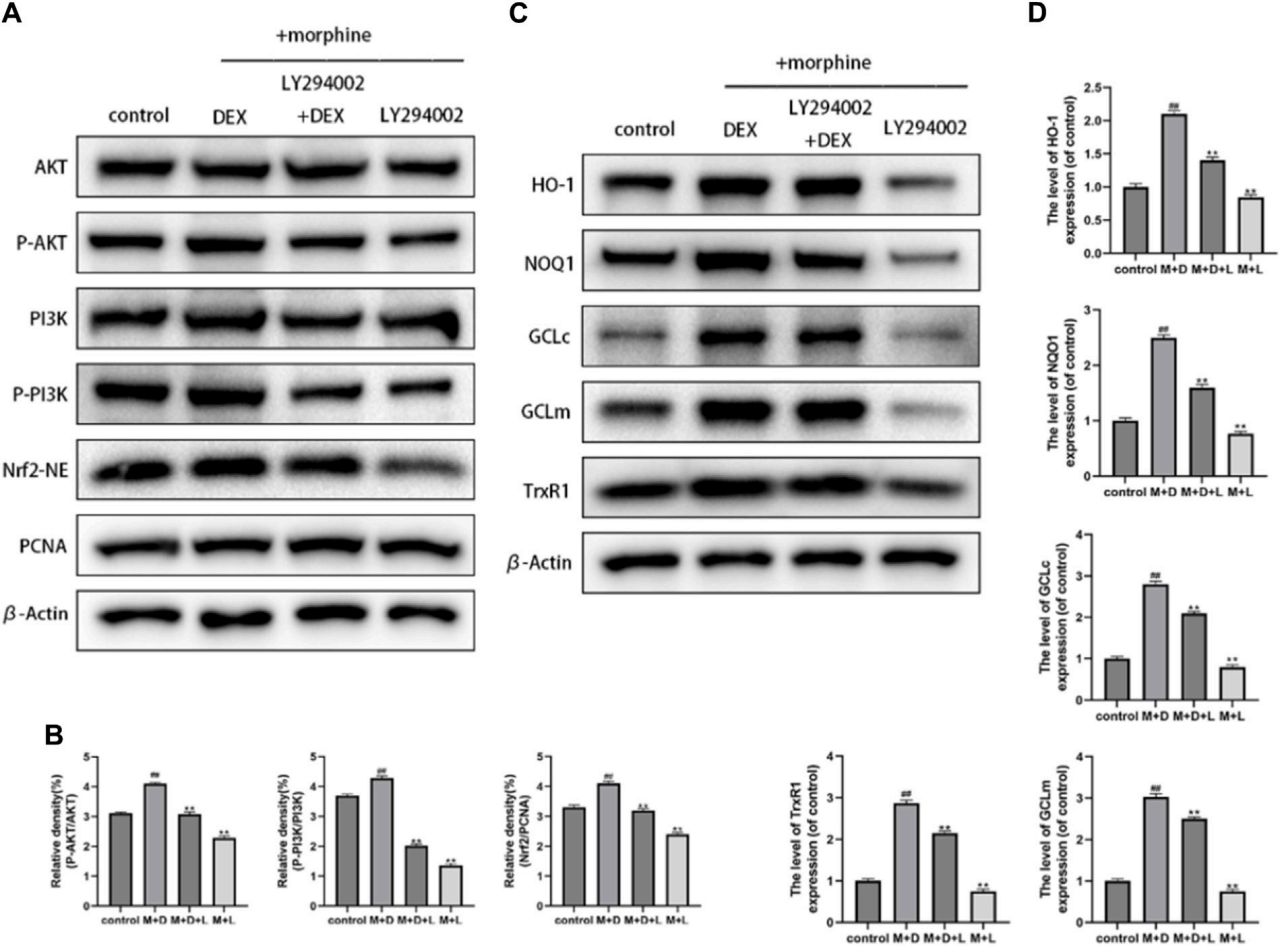
Effects of DEX on the PI3K/Akt/Nrf2 signaling pathway and downstream antioxidant enzymes **(A,B)** Immunoblot demonstrating the degree of PI3K and AKT phosphorylation. The osteoblasts that had received different treatments for Nrf2-NE levels using Western blotting. **(C,D)** The protein expression of GCLc, NQO1, GCLm, HO-1, and TrxR in the osteoblasts after the aforementioned treatment is displayed. Data were evaluated as mean +SD. *p < 0.05, **p < 0.01 compared to the control group; #*p* < 0.05, ##*p* < 0.01, compared to the morphine stimulation group, *n* = 3.

Recent research suggests that the Nrf2 function involves the PI3K/Akt signaling pathway. The activation of downstream targets like Akt and Nrf2 was suppressed using a particular PI3K inhibitor (LY294002) to investigate the mechanism by which DEX upregulates Nrf2 nuclear localization. LY294002 at 10 M significantly reduced the expression of phosphorylated Akt, phosphorylated PI3K, and nuclear Nrf2, as seen in Figures 6A, B. Furthermore, DEX and LY294002 together significantly reduced the increased Akt, PI3K phosphorylation, and Nrf2 nuclear expression caused by DEX at 1 M, showing that DEX-mediated Nrf2 activation was closely related to the PI3K/Akt signaling pathway. The corresponding changes in the gene expression of antioxidant enzymes by the PI3K blocker were discovered to comprehend the protective effectiveness of DEX against morphine-induced oxidative damage through the PI3K/Akt/Nrf2 pathway. The results demonstrated that co-treatment with DEX and LY294002 significantly reduced the expression levels of GCLc, GCLm, NQO1, and TrxR, and HO-1compared to DEX alone (Figures 6C, D).

### 3.7 DEX mitigates morphine-induced delayed bone healing *in vivo*

**FIGURE 7:**
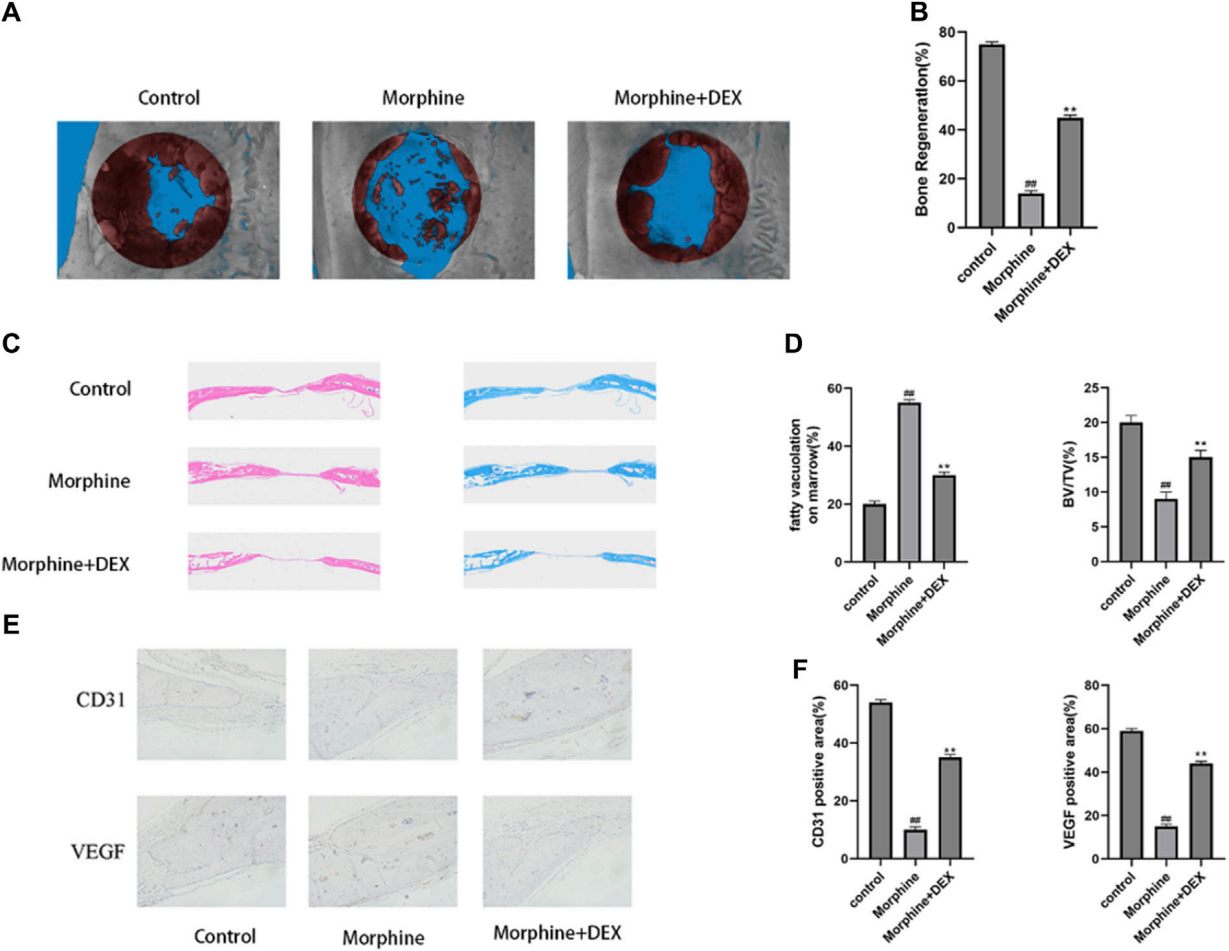
DEX slows down the delayed bone repair caused by morphine *in vivo*. **(A,B)** 12 weeks following surgery, a micro-CT examination of bone repair in calvarial defects was performed. **(C)** H&E staining and Masson’s staining of calvarial bone. **(D)** Quantitative analysis of fatty vacuolation on marrow in HE and BV/TV in Masson staining. **(E,F)** Calvarial tissue was immunohistochemically stained for VEGF and CD31, and the IHC results were quantified. Data were evaluated as mean +SD. *p < 0.05, **p < 0.01 compared to the control group; #*p* < 0.05, ##*p* < 0.01, compared to the morphine stimulation group, *n* = 3.

The essential size calvarial defect in the rats model was created by surgery to examine the preventive impact of DEX against delayed bone healing progression *in vivo*. Micro-CT scans of the morphine group showed minimal new bone growth compared with the control group. In mice treated with DEX, this behavior was, however, less severe (Figures 7A, B). Sections stained with H&E and Masson’s revealed that the morphine group had negligible new bone development. Contrarily, a lot of bone growth was seen in the DEX group (Figures 7C, D). Immunohistochemical examination of VEGF and CD31 was carried out to better understand osteogenesis and angiogenesis (Figure 7E). Representative IHC staining in the morphine group did not show any significant positive staining for VEGF or CD31. The DEX group had more pronounced VEGF and CD31 positive brown staining. These findings were also validated by quantitative analysis (Figure 7F). These results showed that DEX therapy given in combination might enhance neovascularization and bone growth in rats.

## 4 Discussion

The common class of analgesic drugs known as opioids are used to treat both acute and chronic pain that is mild to severe (Corder et al., 2018). Evidence indicates that opioid users have a 55.1% higher risk of fractures. The exact mechanism is thought to be connected to opioid central nervous system side effects, which lower bone density. The secondary cause may be opioid endocrine effects (release of inhibitory hormones like prolactin and growth hormone, hypogonadism), or a direct impact on bone cells (King et al., 2007; Oladeji et al., 2021). Our goal was to research the effects of morphine exposure on Osteoblasts due to the disagreement around morphine’s link with oxidative stress in the present literature and the paucity of information on osteoblasts. The nuclear factor-2 erythroid-related factor (Nrf2), the pathway’s main transcription factor, is responsible for controlling the primary protective mechanisms against oxidative and electrophilic stresses (Ma, 2013). Indeed, it plays a vital role in many physiological functions, including inflammation and mitochondrial function, and it maintains redox homeostasis through endogenous antioxidant mechanisms (Chen, 2022). Numerous genes, including peroxiredoxins, and glutathione peroxidases, thioredoxin reductase 1 (TXNRD1), have been discovered as Nrf2 targets. Currently, more than 250 genes are known to be Nrf2 targets (Gao et al., 2020).

Dex is an agonist of the α-2 receptor that has analgesic, sedative, and anti-sympathetic properties (Lee, 2019). Dex preconditioning is protective against the IRI of important organs such as the brain, liver, heart, kidney, and lung in both *in vitro* and *in vivo* tests (Sun et al., 2019; Tang et al., 2020). Only a small number of research, nevertheless, have looked at how Dex postconditioning affects osteoblasts. Our research proved that Dex postconditioning effectively decreased oxidative stress, avoided apoptosis, and safeguarded osteoblasts. To promote apoptosis in osteoblasts during the *in vitro* experiment, we employed morphine as a ROS source in this work. DEX administration was shown to dramatically lower the levels of cyto and C-caspase3. Additionally, in the investigation at hand, we found that Dex therapy enhanced the expression of the proteins OCN, RUNX2, and COL1A1 that are associated with osteogenesis. This improvement was accompanied by an increase in the osteogenic phenotype of ALP and ARS.

As a crucial transcription factor, Nrf2 is associated with stimulating the expression of downstream genes for a variety of antioxidant enzymes, including SOD, CAT, GPx, and HO-1 (Wang et al., 2021). In the critical size calvarial defect model systems, a variety of Nrf2-activating substances have demonstrated positive benefits, such as lowering inflammatory indicators, oxidative stress, and apoptosis and enhancing synaptic and mitochondrial function (Zhang et al., 2022). Enzymes involved in antioxidant/phase II detoxification contribute to protection by regulating the intracellular redox state (Li et al., 2008). The initial stepwise breakdown of heme is catalyzed by HO-1, producing powerful antioxidants such as free iron, carbon monoxide, and biliverdin (Gao et al., 2022). NQO1 detoxifies reactive quinones to its less harmful hydroquinones, protecting cells from oxidative stress (Ross and Siegel, 2021). TrxR is a homodimeric flavin enzyme that reduces oxidized thioredoxins through NADPH. It contains selenocysteine (Xu et al., 2022). In this work, an efficient antioxidant defense mechanism is provided by the overexpression of TrxR, NQO1, HO-1, and GCLs in response to DEX.

It has been proposed that the Nrf2 upstream regulator is the PI3K/Akt pathway (Liu et al., 2020). According to the results of the current investigation, Nrf2 nuclear accumulation and PI3K/Akt activation were trending in the same direction. Additionally, the DEX-induced production of Nrf2 and its downstream genes was effectively stopped by the PI3K inhibitor LY294002. These results show that DEX increases the nucleus localization of Nrf2, which is facilitated by activating the PI3K/Akt signaling pathway, and therefore stimulates the production of a group of antioxidant/phase II detoxification enzymes.

There is growing evidence that two major pathways can activate Nrf2 (Best et al., 2018). First, it specifically targets the Nrf2/Keapl complex by oxidizing the cysteine residues in Keapl, which modifies Keapl’s conformation and causes the Nrf2-Keapl complex to dissociate (Zhao et al., 2018). The second mechanism involves the activation of upstream kinases such as mitogen-activated protein kinases (MAPKs), PI3K/Akt, AMP-activated protein kinase (AMPK), and protein kinase C (PKC). This causes Nrf2 to be phosphorylated and translocated into the nucleus, which encourages the expression of the antioxidant enzyme (Zhuang et al., 2019). According to the findings of the Western blot investigation, DEX activated Nrf2 and consequent downstream antioxidant enzymes through a twofold mechanism that involved the disruption of the Nrf2-Keapl complex and the PI3K/Akt axis. This allowed Nrf2 to be released and go into the nucleus, where it initiates the transcription of antioxidant genes. Our results offer the first proof that DEX may counteract the oxidative stress caused by morphine by triggering the antioxidant defense mechanism that is driven by Nrf2 and that DEX upregulates and activates Nrf2 through the PI3K/Akt pathway.

The result of the present study should be interpreted with some limitations. 1) In the preparation of the animal model for this study, a intraperitoneal dose of 50 mg/kg pentobarbital sodium was used to make rats lose consciousness. Therefore, the combined effect of dexmedetomidine and morphine was obtained under the premise of using pentobarbital sodium, and the combined effect of the two drugs under other anesthesia methods needs to be further explored. 2) There are multiple pathways involved in antioxidant activity, and studies have shown that dexmedetomidine also has an effect on other pathways. This experiment only studied the Nrf2-pI3k pathway. 3) Dexmedetomidine and morphine are commonly used drugs in clinical anesthesia, and the combined use of the two drugs still needs clinical validation for their impact on bone healing.

## 5 Conclusion

The above results give empirical support for the hypothesis that DEX prevents morphine-induced oxidative injury by enhancing the Nrf2-mediated antioxidant system and PI3K/Akt pathway. According to molecular docking research, Dex displayed substantial interactions with PI3K, Akt, and Nrf2-keapl through hydrogen bonds and van der Waals forces. The first time, the proof was shown that DEX exerted safeguarding properties by activating the antioxidant defense mechanism regulated by PI3K/Akt/Nrf2 in morphine-induced oxidative stress.

## Data Availability

The original contributions presented in the study are included in the article/supplementary material, further inquiries can be directed to the corresponding authors. The original raw data can be found here: https://pan.baidu.com/s/1bL7jX0Ba7E00Ly6Kzaq7vQ, password: 2t54.
